# Determining dimensions of job satisfaction in healthcare using factor analysis

**DOI:** 10.1186/s40359-022-00941-2

**Published:** 2022-10-27

**Authors:** Dimitris Karaferis, Vassilis Aletras, Dimitris Niakas

**Affiliations:** 1 Department of Health Economics, Medical School, National and Kapodistrian University of Athens, 75, M. Assias Street, 11527 Athens, Greece; 2 Department of Business Administration, University of Macedonia, Thessaloniki, Greece

**Keywords:** Healthcare, Job satisfaction, Exploratory factor analysis, Principal component analysis, Confirmatory factor analysis, Greece

## Abstract

**Background:**

Job satisfaction in health care has a great impact as it affects quality, productivity, effectiveness, and healthcare costs. In fact, it is an indicator of the well-being and quality of life of the organization’s employees, as it has been variously linked with increased performance and negatively to absenteeism and turnover. Better knowledge of healthcare employees’ job satisfaction and performance can directly contribute to the quality of the services provided to patients and is critical for the success of organizations.

**Methods:**

The Cronbach’s alpha coefficient, split-half reliability, exploratory factor and confirmatory factor analysis were employed to assess the reliability and validity of JSS.

**Results:**

Six underlying dimensions were extracted (benefits and salary, management’s attitude, supervision, communication, nature of work, and colleagues’ support). Internal consistency reliability was satisfactory since Cronbach’s alpha for the overall scale was 0.81 and for the various dimensions ranged from 0.61 to 0.81, respectively. Exploratory factor analysis showed a KMO value of 0.912. The confirmatory factor analysis indicated good fit: SRMR = 0.050, RMSEA = 0.055, IFI = 0.906 and CFI = 0.906.

**Conclusion:**

Job satisfaction is a multidimensional construct that encompasses different facets of satisfaction. There is a lack of consensus as to which factors are more important and a researcher may find satisfaction with some factors while at the same time dissatisfaction with others. Our findings are significant for improving our understanding of the nature and assessment of job satisfaction in the Greek healthcare context, providing a more stable ground in a rapidly changing environment. A short JSS developed that could be much more widely used in the future.

## Introduction

As employee knowledge and skills are intangible assets of any service organization,

employee satisfaction has become an issue of utmost importance. It has been defined as the positive emotional state resulting from the evaluation of one’s work or work experience [1]. Hoppock [2] was the first who brought forth the concept of job satisfaction in limelight and described it as “the employees’ subjective reflections or subjective feelings about their working conditions and working environment”. Since then, many researchers have recognized that satisfied employees are a key asset to an organization [3]. While the importance of job satisfaction is generally recognized, additional and ongoing investigations of satisfaction levels are necessary as external conditions and societal values are constantly changing. In this respect, job satisfaction has a significant role in the operation and performance of organizations.

An essential prerequisite for the development and long-term success of an organization is in fact the utilization of employee’s capabilities and the improvement of their working conditions [4]. The degree of job satisfaction is actually the overall level of satisfaction on a number of different dimensions of work and affects the behavior of employees that, in turn, impacts upon organizational functioning [5–7]. Swamy et al. [3] stated that satisfied employees are the key asset of an organization. Therefore, the issue of job satisfaction is very important especially for non-profit public organizations like hospitals, which are essential for a country’s provision of healthcare services and the population itself.

Employee satisfaction also affects patient satisfaction. As patients are the external customers and employees are the internal customers of the organization they form the current working environment and are willing to cooperate with the community to achieve organizational goals. Previous studies have documented associations between job fulfillment of health workforce and patient contentment with the type of health care services provided in health care facilities [8, 9]. Moreover, there seems to exist a positive correlation between the increase in job satisfaction and quality of care [10, 11]. Conversely, a low level of job satisfaction would create negative behaviors, including absenteeism, grievances, high level of stress, turnover, exhaustion, low morality, worse patient-provider ratios, longer wait times, psychological distress and increased medical errors [12–14].

Hospital managers have responsibilities to both patients and staff. It has been suggested that if you want to attain higher job productivity and efficiency, you should comprehend the domains of work which are decisive for job satisfaction amongst healthcare providers. In order to get employees contented with their job; the underlying factors that influence job satisfaction in that particular facility must be examined to guide proper managerial action [15, 16].

## Measurement of job satisfaction

Due to its importance, a wide range of instruments have been designed to quantify and conceptualize job satisfaction during the past decades. They were developed to capture the entirety of various aspects of job satisfaction be it personal, social, environmental, organizational, and the nature of the job itself. A valuable and widely used measure of job satisfaction is the Job Satisfaction Survey (JSS) that was originally developed by Spector [17]. JSS provides sufficient reliability and validity and is available for researchers free of charge for use for non-commercial purposes. The instrument contains 36 items expressed on a Likert scale measuring nine dimensions of job satisfaction, as mentioned below:

- Pay includes salaries and wages. Unfair distribution can negatively affect employees’ emotions and therefore their behavior in the organization [18].
- Promotion is an important aspect of a employee’s career. It refers to progression to a higher position with more challenges, authority and responsibilities [19]. Only a meritocratic promotion system with evaluation conditions known in advance can lead to satisfaction.
- Fringe Benefits, can be financial or non-financial compensations. Financial compensations consist of direct (e.g. bonuses) and indirect compensation (e.g. retirement plans). Non-financial compensations consist of the job itself (e.g. autonomy), job environment (e.g. working conditions) and workplace flexibility (e.g. part-time work) [20].
- Contingent Rewards, are referred to as promises and exchanges of rewards and recognition for good work. Is a valuable tool for motivating employees because they want to be paid well for the job they perform both for their self-esteem and as useful means of a living [21].
- Supervision, is defined as the perception of employees regarding the support received from supervisors in an organization besides coworkers. Usually, employees are satisfied when they are supported to achieve their goals [22].
- Operating Procedures, are described as steps of finishing tasks that have to follow a certain standard based on regulations, provincial laws, policies, procedures and standards. Inadequacy of equipment and resources, lighting, ventilation, and cleanliness can result in a stressful work environment that leads to job dissatisfaction among employees [23].
- Co-workers, are referred as people working in an organization (besides supervisors). Employees with the same values, attitudes and philosophies can improve satisfaction in an organization [24]. Support from colleagues can enhance job satisfaction and decrease job stress and burnout.
- Nature of Work, is defined as the variability of the given work. It refers to the daily and non-daily tasks carried out as part of the job scope and includes job challenges, feedback, autonomy, and skill variety [25]. Further, this can increase the motivational level of employees which will ultimately raise their internal happiness of employees, and the internal happiness will cause satisfaction.
- Communication, is referred as informing the current employees. Communication between supervisors or the managerial level with employees consistently enables managers to know whether their staff is satisfied and happy with its employment or not [26]. There is a positive association between communication and job satisfaction. Effective communication at the workplace is essential in ensuring organizational objectives, social support.

Every dimension incorporates four items. Several previous studies have shown that JSS has high internal consistency and validity [27, 28].

## Objectives

This research aimed to explore (a) the underlying factorial structure of the JSS when applied to Greek hospital employees, (b) its psychometric properties. Undoubtedly, job satisfaction is a complex concept, so there is always a need to research this phenomenon and related factors to explore the development of optimal human resources strategies in the context of healthcare institutions. Moreover, there is a compelling need for developing constructs in the field of management rather than adapting the constructs that have been developed already.

## Materials and methods

### Research instrument translation and adaptation

The JSS has been translated in several languages and found to be valid and reliable among different categories of employees. Spector’s original JSS tool was translated into the Greek language and adapted by Tsounis and Sarafis [27] to be administered to employees of the Greek Therapy Centre for Dependent Individuals. In this context, the JSS was translated into Greek using the forward-backward translation process. Firstly, the original English of the JSS was translated into the Greek language by two experienced translators. The assessment of forwarding translation drafts was performed by two other researchers who worked independently and asked to review each translated item and choose the most adequate in terms of clarity, common language, and cultural diversity. Secondly, a retranslation of the agreed Greek text to the English language was held by a researcher who had not previously seen the original version. Thirdly, the backward translation was compared with the original version of the survey, and judgments about potential inaccuracies were made by two other researchers. Finally, the resulting differences were checked by another scientist who made the necessary adjustments.

The reliability and validity of the tool has been documented worldwide in a variety of settings. Reliability coefficients of prior and current research are presented in Table 1. The measures whose Cronbach’s Alpha exceeds 0.6 are considered to be the reliable ones and indicates an acceptable level of reliability [29–31]. Schmitt [32] has suggested that there is no general level (such as 0.7) where alpha becomes acceptable. In reality, a key feature of the alpha coefficient is that it is highly dependent on the number of items involved. Thus, if we wish to reduce the items in our survey (e.g. EFA), because of this, a small number of well-correlated items may have a fairly low alpha coefficient. Conversely, since there are more items, the value of alpha can be quite high despite the low correlation between many of these items. Addionnaly, Ursally [33] showed that important differences in the values of Cronbach Alpha are possible due to indirect influences from external factors - respondents’ age, gender, level of study, religiousness, rural/urban living, and survey type of the research subject for the participants to the survey [34, 35].

**Table 1:** Job Satisfaction Survey Dimensions, Descriptions and Cronbach’s Alpha

Dimensions	Descriptions of Dimensions	Items	Spector, 1985	Greek Sample Tsounis & Sarafis, 2018	Present study*
Pay	Pay and remuneration	1, 10, 19, 28	0.75	0.62	0.66
Promotion	Promotion opportunities	2, 11, 20, 33	0.73	0.67	0.65
Supervision	Immediate supervisor	3, 12, 21, 30	0.82	0.87	0.81
Fringe Benefits	Monetary and nonmonetary fringe benefits	4, 13, 22, 29	0.73	0.73	0.68
Contingent rewards	Appreciation, recognition and rewards for good work	5, 14, 23, 32	0.76	0.71	0.74
Operating procedures	Operating policies and procedures	6, 15, 24, 31	0.62	0.48	0.41
Co-workers	People you work with	7, 16, 25, 34	0.60	0.67	0.62
Nature of work	Job tasks themselves	8, 17, 27, 35	0.78	0.74	0.62
Communication	Communication within the organization	9, 18, 26, 36	0.71	0.71	0.64
Overall Satisfaction		All items	0.91	0.87	0.89

* To calculate Cronbach’s Alpha coefficients, we took into consideration the creator’s suggestion to reverse 19 of the statements (2,4,6,8,10,12,14,16,18,19,21,23, 24,26,29,31,32,34,36)

Prior reliability analysis of the translated and adapted Greek version of the instrument seems to have some issues. First of all, one facet of job satisfaction had Cronbach’s alpha below 0.6 (i.e., 0.48 for “Operating procedures”). Second, the JSS was applied and evaluated on 239 employees of various specialties in drug addiction treatment of one only medical center with common structure. This implies that the sample size might be rather small for factor analysis and that its findings might not even be generalizable [31, 36].

Additionally for this research, Split-half reliability analysis (Table 2) was assessed by dividing the instrument into two halves; Part 1: consisted of the first 18 items, and Part 2: consisted of the remaining 18 items of the scale. The findings showed that JSS had good split-half reliability as assessed through the Guttman Split-Half Coefficient (0.77).

**Table 2:** Split-Half reliability analysis

Cronbach’s Alpha		Part 1	Value	0.81
			N of Items	18^a^
		Part 2	Value	0.83
			N of Items	18^b^
		Total N of Items		36
Correlation Between Forms				0.63
Spearman-Brown Coefficient			Equal Length	0.77
			Unequal Length	0.77
Guttman Split-Half Coefficient				0.77

a. The items are: Q1, Q2, Q3, Q5, Q7, Q9, Q11, Q13, Q15, Q17, Q20, Q22, Q25, Q27, Q28, Q30, Q33, Q35.

b. The items are: Q4, Q6, Q8, Q10, Q12, Q14, Q16, Q18, Q19, Q21, Q23, Q24, Q26, Q29, Q31, Q32, Q34, Q36.

## Research design and procedure

The survey was carried out in the region of Attiki with its capital Athens, with around 3.75 million inhabitants or approximately 35% of the total Greek population. The 1st Regional Health Authority of Attica has the responsibility for 27 public hospitals. Our survey was conducted between July 2019 and December 2020 in thirteen of those who provided healthcare services to 438,745 patients. The main criteria for the selection of these hospitals were (Table 3): (a) the categories of hospitals; for this reason, the survey was introduced into four different categories (general, pediatric, maternity, oncology), (b) a large number of different clinics, (c) hospitals with a large number of beds but without ignoring the role of smaller hospitals, (d) the large number of patients treated in these hospitals, (e) the large number of health care employees who work in these hospitals, and (f) the necessary approval of the research by hospital committees.

The researchers distributed the printed questionnaire along with a consent form to the participants in person at their workplaces. They were adults (over 18 years), health care professionals belonging to medical, nursing, administrative, and technical departments serving public healthcare. The main aim of selecting employees from various fields is to get the opinions of a diverse group of people so that the results can be generalized on s vast group of the overall population. They had worked for more than six months in the respective hospital facilities at the time of the research and consented to the study. The study excluded interns, volunteers, and those declining to consent to the study. The participants had one week to complete the questionnaire. All employees had the right to refuse or discontinue their participation in the survey at any time. The researcher guaranteed the anonymity and confidentiality of all data collected. We remained considerate of the names, safety, and well-being of participants, and also the organizations remained anonymous by using codes, such as H01, H02, and so on (Table 3). Finally, of the 4,000 questionnaires distributed, 3,278 (81.95%) were returned.

**Table 3:** The population of research per hospital and category. Distributed Questionnaires and Response Rate

	Period of research						Professional Category										
	**2019**		**2020**				**Doctors**		**Nurses**		**Other Health Professionals**		**Overall Sample**		**Number**	**Number**	**Days of**
**Hospital**	**3Q**	**4Q**	**1Q**	**2Q**	**3Q**	**4Q**	**( n )**	**( % )**	**( n )**	**( % )**	**( n )**	**( % )**	**( n )**	**( % )**	οf Beds*	οf Patients*	Hospitalization*
H 01	X	X	X				108	13.45%	132	7.60%	43	5.82%	283	8.64%			
H 02	X	X	X	X			168	20.92%	290	16.71%	158	21.38%	616	18.80%			
H 03			X	X	X	X	29	3.61%	99	5.70%	74	10.01%	202	6.17%			
H 04		X	X	X	X	X	90	11.21%	210	12.10%	64	8.66%	364	11.11%			
H 05	X	X	X				59	7.35%	89	5.13%	62	8.39%	210	6.41%			
H 06			X	X	X	X	51	6.35%	122	7.03%	69	9.34%	242	7.39%			
H 07		X	X	X			61	7.60%	181	10.43%	39	5.28%	281	8.58%			
H 08		X	X	X			87	10.83%	90	5.18%	32	4.33%	209	6.38%			
H 09		X	X	X			36	4.48%	119	6.85%	57	7.71%	212	6.47%			
H 10		X	X	X			45	5.60%	111	6.39%	50	6.77%	206	6.29%			
H 11		X	X				29	3.61%	136	7.83%	36	4.87%	201	6.14%			
H 12		X	X	X	X		32	3.99%	132	7.60%	38	5.14%	202	6.17%			
H 13			X				8	1.00%	25	1.44%	17	2.30%	50	1.53%			
[1] Hospitals of research						(n = 13)	803	24.50%	1,736	52.96%	739	22.54%	3,278	100%	6,511	438,745	1,443,660
[2] Hospitals in 1st Regional Health Authority of Attica						(n = 27)	6,277**	31.71%	8,174**	41.29%	5,345**	27.00%	19,796**	100%	8,860	567,817	1,912,488
[3] Hospitals in the National Health System						(n = 127)									36,441	2,160,596	7,343,348
[4] Percentages of Hospitals of research / Hospitals of Attica						= [1] / [2]			12.79%		21.24%		13.83%		16.56%	73.49%	77.27%
[5] Percentages of Hospitals of research / NHS						= [1] / [3]									17.87%	20.31%	19.66%
[6] Distributed Questionnaires of research							1,000	25.00%	2,000	50.00%	1,000	25.00%	4,000	100%			
[7] Response Rate						= [1] / [6]		80.30%		86.80%		73.90%		81.95%			

Notes: * Data of year 2020 ** Data of year 2016

X = research conduct

### Statistical analysis

The data collected were analyzed using SPSS software (version 24.0). The mean (M) and standard deviation (SD) of each JSS item were determined. The reliability coefficient was examined. As a rule of thumb, values of Cronbach’s α ≥ 0.6 are thought to be acceptable [31]. Validity was evaluated using convergent and discriminant validity, as well as factor analysis consisting of exploratory factor analysis (EFA) and Confirmatory Factor Analysis (CFA).

Exploratory factor analysis (EFA) was conducted by utilizing principle component analysis (PCA) with the varimax rotation method, which had applied an Eigenvalue of > 1 for this purpose. For EFA we used the Kaiser-Meyer-Olkin (KMO) statistic was employed to assess whether the sample data are suitable for factor analysis. According to Kaiser [37], a value above 0.5 is considered acceptable; between 0.5 and 0.7 is moderate; between 0.7 and 0.8 is good; between 0.8 and 0.9 is very good; and 0.9 and above is superb. Also, Bartlett’s Test was applied to verify if the data was appropriate for factor analysis and indicated that correlations between items were sufficiently large for PCA. Retained and excluded factors were also explored visually on a screen plot along with the parallel analysis. Many studies reported that factor loadings should be greater than 0.5 for better results [38–40]. Principal Component Analysis was chosen as the suitable extraction method for obtaining the initial factor solution and reducing the number of factors. PCA is a robust method that is psychometric and less complex conceptually than other methods and is also preferred because it resembles many aspects of discriminate analysis. Varimax rotation of the factors was also applied to produce the factor structure. The advantage of Varimax rotation is that maximizes distribution within the factors, thus introducing a small number of variable loads and more easily interpretable factor clusters into each factor load. Cross-loaded statements also were deleted [38–41].

After using EFA to identify the factor structure present in a set of variables, the model fit was then assessed by using Confirmatory Factor Analysis (CFA). A CFA with a maximum likelihood method (ml) in AMOS (version 24.0) was also performed. The fit of the CFA model was assessed using the incremental and absolute indexes, namely: the comparative fit index (CFI), incremental fit index (IFI), the standard mean root square residual (SRMR) and the root mean square error of approximation (RMSEA). The following cut-off values were assumed: CFI, and IFI ≥ 0.900, SRMR and RMSEA ≤ 0.800 [42, 43].

## Results

### Study sample

Among the sample participants 612 (18.67%) were male and 2,666 (81.33%) were female. Regarding their age, 1.49% was under 25 years old, 15.86% were 26–35, 33.25% were between 36 and 45, 38.16% between 46 and 55. The remaining 11.23% were older than 56 years. As far as the educational level is concerned, the majority was university graduates (59.55%), 19.37% had post-graduate studies, only 1.53% had compulsory education and the remaining 19.55% had secondary education. Concerning employment status, the majority worked as permanent staff (80.99%). As regards length of service, 19.37% had less than 5 years, 11.90% of study participants had worked from 6 to 10 years, 17.63% from 11 to 15 years, 22.45% from 16 to 20 years, while 28.65% had worked for more than 20 years. With respect to income, the majority of employees stated that they managed without having much money left aside (see Table 4).

**Table 4:** Sociodemographic characteristics of the sample per professional category

		Professional Categories							
**Characteristics**		**Doctors**		**Nurses**		**Other Health Professionals**		**Overall Sample**	
		**N = 803**	**%**	**N = 1,736**	**%**	**N = 739**	**%**	**N = 3,278**	**%**
**Gender**	Male	294	36.61%	150	8.64%	168	22.73%	612	18.67%
	Female	509	63.39%	1,586	91.36%	571	77.27%	2,666	81.33%
**Age**	< 25 years	5	0.62%	32	1.84%	12	1.62%	49	1.49%
	26–35 years	236	29.39%	243	14.00%	41	5.55%	520	15.86%
	36–45 years	273	34.00%	612	35.25%	205	27.74%	1,09	33.25%
	46–55 years	210	26.15%	723	41.65%	318	43.03%	1,251	38.16%
	56 > years	79	9.84%	126	7.26%	163	22.06%	368	11.23%
**Marital**	Married	385	47.95%	1,17	67.40%	499	67.52%	2,054	62.66%
**Status**	Single	393	48.94%	431	24.83%	152	20.57%	976	29.77%
	Divorced	24	2.99%	124	7.14%	62	8.39%	210	6.41%
	Widowed	1	0.12%	11	0.63%	26	3.52%	38	1.16%
**Level of**	Compulsory	0	0.00%	7	0.40%	43	5.82%	50	1.53%
**Education**	Secondary	0	0.00%	313	18.03%	328	44.38%	641	19.55%
	Bachelor	559	69.61%	1,099	63.31%	294	39.78%	1,952	59.55%
	Master’s / PhD	244	30.39%	317	18.26%	74	10.01%	635	19.37%
**Employment**	Permanent	425	52.93%	1,59	91.59%	640	86.60%	2,655	80.99%
**Status**	Temporary	378	47.07%	146	8.41%	99	13.40%	623	19.01%
**Professional**	< 5 years	290	36.11%	221	12.73%	124	16.78%	635	19.37%
**Experience**	6–10 years	158	19.68%	158	9.10%	74	10.01%	390	11.90%
	11–15 years	114	14.20%	376	21.66%	88	11.91%	578	17.63%
	16–20 years	135	16.81%	457	26.32%	144	19.49%	736	22.45%
	20 > years	106	13.20%	524	30.18%	309	41.81%	939	28.65%
**Economic Situation**	I cannot cope with my financial obligations	2	0.25%	70	4.03%	55	7.44%	127	3.87%
	I manage financially with great difficulties	108	13.45%	716	41.24%	363	49.12%	1,187	36.21%
	I manage financially but I do not have much left aside	570	70.98%	871	50.17%	274	37.08%	1,715	52.32%
	I am financially comfortable	105	13.08%	31	1.79%	25	3.38%	161	4.91%
	I do not know / I do not answer	18	2.24%	48	2.76%	22	2.98%	88	2.68%

## Normality analysis

The Kolmogorov-Smirnov and Shapiro-Wilk normality tests were performed and showed that the data was not normally distributed (p < 0.05).

## Descriptive statistics results

Descriptive statistics for the items of the questionnaire are shown in Table 5. The results indicate that the minimum value of the items is 1 while the maximum is 6.

**Table 5:** Surveys questions and descriptive statistics (N = 3,278)

Item no.	Survey Questions	Min.	Max.	Mean		Median	Percentiles			Std. Deviation	Variance Statistic	Sum		
				**Statistic**	**Std. Error**		**25**	**50**	**75**					
JS 1	I feel I am being paid a fair amount for the work I do	1	6	2.46	0.021	2.00	2.00	2.00	3.00	1.193	1.424		8049	
JS 2	There is really too little chance for promotion on my job	1	6	2.72	0.022	3.00	2.00	3.00	3.00	1.260	1.587		8905	
JS 3	My supervisor is quite competent in doing his/her job	1	6	4.77	0.018	5.00	4.00	5.00	5.00	1.050	1.103		15,649	
JS 4	I am not satisfied with the benefits I receive	1	6	2.93	0.021	3.00	2.00	3.00	4.00	1.202	1.444		9598	
JS 5	When I do a good job, I receive the recognition for it that I should receive	1	6	3.26	0.022	3.00	2.00	3.00	4.00	1.284	1.649		10,678	
JS 6	Many of our rules and procedures make doing a good job difficult	1	6	2.71	0.019	3.00	2.00	3.00	3.00	1.065	1.135		8870	
JS 7	I like the people I work with	1	6	5.10	0.013	5.00	5.00	5.00	6.00	0.733	0.538		16,717	
JS 8	I sometimes feel my job is meaningless	1	6	3.90	0.023	4.00	3.00	4.00	5.00	1.344	1.806		12,790	
JS 9	Communications seem good within this organization	1	6	4.00	0.019	4.00	4.00	4.00	5.00	1.097	1.204		13,114	
JS 10	Raises are too few and far between	1	6	1.73	0.019	1.00	1.00	1.00	2.00	1.070	1.144		5657	
JS 11	Those who do well on the job stand a fair chance of being promoted	1	6	2.43	0.021	2.00	1.00	2.00	3.00	1.230	1.512		7956	
JS 12	My supervisor is unfair to me	1	6	4.71	0.020	5.00	4.00	5.00	5.00	1.133	1.284		15,448	
JS 13	The benefits we receive are as good as most other organizations offer	1	6	2.28	0.020	2.00	1.00	2.00	3.00	1.154	1.332		7488	
JS 14	I do not feel that the work I do is appreciated	1	6	2.89	0.022	3.00	2.00	3.00	4.00	1.237	1.530		9483	
JS 15	My efforts to do a good job are seldom blocked by red tape	1	6	3.45	0.023	4.00	3.00	4.00	4.00	1.292	1.670		11,308	
JS 16	I find I have to work harder at my job because of the incompetence of people I work with	1	6	3.80	0.022	4.00	3.00	4.00	5.00	1.276	1.629		12,458	
JS 17	I like doing the things I do at work	1	6	4.87	0.015	5.00	5.00	5.00	5.00	0.877	0.769		15,979	
JS 18	The goals of this organization are not clear to me	1	6	3.15	0.021	3.00	2.00	3.00	4.00	1.190	1.415		10,311	
JS 19	I feel unappreciated by the organization when I think about what they pay me	1	6	2.33	0.020	2.00	2.00	2.00	3.00	1.164	1.356		7636	
JS 20	People get ahead as fast here as they do in other places	1	6	2.30	0.020	2.00	1.00	2.00	3.00	1.148	1.318		7547	
JS 21	My supervisor shows too little interest in the feelings of subordinates	1	6	4.29	0.022	4.00	4.00	4.00	5.00	1.269	1.610		14,049	
JS 22	The benefit package we have is equitable	1	6	2.79	0.021	3.00	2.00	3.00	4.00	1.175	1.380		9137	
JS 23	There are few rewards for those who work here	1	6	2.72	0.021	3.00	2.00	3.00	3.00	1.192	1.422		8909	
JS 24	I have too much to do at work	1	6	2.14	0.016	2.00	2.00	2.00	3.00	0.897	0.805		7001	
JS 25	I enjoy my coworkers	1	6	4.81	0.016	5.00	4.00	5.00	5.00	0.898	0.807		15,760	
JS 26	I often feel that I do not know what is going on with the organization	1	6	3.82	0.022	4.00	3.00	4.00	5.00	1.258	1.582		12,532	
JS 27	I feel a sense of pride in doing my job	1	6	4.82	0.018	5.00	4.00	5.00	5.00	1.013	1.027		15,802	
JS 28	I feel satisfied with my chances for salary increases	1	6	1.98	0.019	2.00	1.00	2.00	2.00	1.107	1.226		6486	
JS 29	There are benefits we do not have which we should have	1	6	2.67	0.021	3.00	2.00	3.00	3.00	1.189	1.414		8736	
JS 30	I like my supervisor	1	6	4.86	0.016	5.00	4.00	5.00	5.00	0.936	0.876		15,941	
JS 31	I have too much paperwork	1	6	2.97	0.023	3.00	2.00	3.00	4.00	1.293	1.671		9742	
JS 32	I don’t feel my efforts are rewarded the way they should be	1	6	2.79	0.020	3.00	2.00	3.00	4.00	1.167	1.362		9132	
JS 33	I am satisfied with my chances for promotion	1	6	2.35	0.021	2.00	1.00	2.00	3.00	1.176	1.383		7715	
JS 34	There is too much bickering and fighting at work	1	6	3.31	0.020	3.00	3.00	3.00	4.00	1.166	1.361		10,855	
JS 35	My job is enjoyable	1	6	3.75	0.021	4.00	3.00	4.00	4.00	1.181	1.395		12,293	
JS 36	Work assignments are not fully explained	1	6	4.17	0.021	4.00	4.00	4.00	5.00	1.187	1.410		13,673	

The highest mean values were found for Item–7 and Item–17 while the lowest ones for Item–10 and Item–28. The average variability of the items around mean values was relatively small.

## Exploratory factor analysis

According to the analysis result, the KMO (Kaiser-Meyer-Olkin) statistic of 0.912 confirmed that the sample used was quite sufficient. We can therefore be confident that the factor analysis fits into our data set. Next, Barlett’s test of sphericity (*χ*^2^ = 31831.572, *df* = 528, *p* = 0.000) demonstrated that the correlation matrix is not an identity matrix, therefore providing justification for the use of factor analysis [37, 44]. In PCA the eigenvalue provides the fraction of the variation accounted for by the corresponding component (eigenvector). We adopted a combined criteria method as suggested by Lings and Greenley [45], and Larose [46] to identify items and factors for inclusion in the final factorial solution. More specifically, to evaluate the factor structures, we used four criteria. First, items factor loadings should be at least equal to or greater than 0.5. Second, a scale should have more than two items or if it has only two they should be strongly correlated. Third, if an item loads more than one dimension and their difference is lower than 0.02, it will be deleted. Moreover, the difference in loadings, equal to or greater than 0.2, implies the item’s inclusion in the dimension with the highest factor load. Finally, in order to maintain an item, it would also have to conceptually match the factor [47–49].

Based on an eigenvalue greater than one, as one eigenvalue represents a significant amount of variation, factors considered in subsequent analyses. Hence, another eigenvalue-based approach was used to examine Cattell’s “scree” plot, by looking for a spot in the plot where it abruptly levels out. By employing both methods, a six-factor model was identified (see Table 6) [50]. The final factorial structure explains 56.23% of the total variance of the dataset. According to the results obtained, the first factor had 23.78% of the total variance, the second factor 11.52%, the third factor 6.64%, the fourth factor 6.30%, the fifth factor 4.17%, and the sixth factor 3.81%. The total variance explanatory rates of the factors after rotation were as follows: 14.13%, 10.53%, 10.49%, 8.19%. 6.92% and 5.97%.

**Table 6:** Eigenvalues and the explained total variance of the extracted factors

	Initial Eigenvalues			Extraction Sums of Squared Loadings			Rotation Sums of Squared Loadings		
Component	Total	% of Variance	Cumulative %	Total	% of Variance	Cumulative %	Total	% of Variance	Cumulative %
1	6.18	23.78	23.78	6.18	23.78	23.78	3.67	14.13	14.13
2	3.00	11.52	35.31	3.00	11.52	35.31	2.74	10.53	24.66
3	1.73	6.64	41.94	1.73	6.64	41.94	2.73	10.49	35.14
4	1.64	6.30	48.24	1.64	6.30	48.24	2.13	8.19	43.34
5	1.09	4.17	52.41	1.09	4.17	52.41	1.80	6.92	50.26
6	1.00	3.81	56.23	1.00	3.81	56.23	1.55	5.97	56.23

Extraction Method: Principal Component Analysis

Varimax rotation was used for the rotation of the original solution as our sampling has a heterogeneous population [51, 52]. Twenty variables were included within six factors. The resulting six factors were: Factor 1 which indicates employees’ benefits and salary includes items: 11, 20, 28, 33. Factor 2 represents the management’s attitude and includes items: 14, 19, 24, 29. Factor 3 supervision and includes items: 3, 12, 21, 30. Factor 4 represents employees’ communication, includes items: 18, 26 and 36. Factor 5 mainly indicates the nature of work and includes items: 17, 27,35 and finally Factor 6 consists colleagues support and includes items: 7 and 25 (Table 7).

**Table 7:** Standardized loadings of items for each factor

			Factor 1	Factor 2	Factor 3	Factor 4	Factor 5	Factor 6
**Item** **no.**	**Questions**	**Dimensions**	**Benefits and Salary**	**Management’s attitude**	**Supervision**	**Communication**	**Nature of work**	**Colleagues Support**
JS 33	I am satisfied with my chances for promotion	Promotion	0.83					
JS 28	I feel satisfied with my chances for salary increases	Pay	0.77					
JS 20	People get ahead as fast here as they do in other places	Promotion	0.76					
JS 11	Those who do well on the job stand a fair chance of being promoted	Promotion	0.72					
JS 24	I have too much to do at work	Operating procedures		0.83				
JS 29	There are benefits we do not have which we should have	Fringe Benefits		0.70				
JS 19	I feel unappreciated by the organization when I think about what they pay me	Pay		0.56				
JS 14	I do not feel that the work I do is appreciated	Contingent rewards		0.54				
JS 3	My supervisor is quite competent in doing his/her job	Supervision			0.83			
JS 30	I like my supervisor	Supervision			0.81			
JS 21	My supervisor shows too little interest in the feelings of subordinates	Supervision			0.77			
JS 12	My supervisor is unfair to me	Supervision			0.74			
JS 26	I often feel that I do not know what is going on with the organization	Communication				0.78		
JS 18	The goals of this organization are not clear to me	Communication				0.68		
JS 36	Work assignments are not fully explained	Communication				0.67		
JS 17	I like doing the things I do at work	Nature of work					0.81	
JS 27	I feel a sense of pride in doing my job	Nature of work					0.75	
JS 35	My job is enjoyable	Nature of work					0.63	
JS 7	I like the people I work with	Coworkers						0.82
JS 25	I enjoy my coworkers	Coworkers						0.81

Notes: (1) The weights of extracted factors from exploratory factor analysis with varimax rotation (weights less than 0.4 are not displayed). (2) Extraction Method: Principal Component Analysis. (3) Factor loading > 0.5

The reliability coefficient Cronbach’s alpha of new construction of scales after application of factor analysis for the overall scale was 0.81 and we concluded that the questionnaire has very good reliability. The results showed that obtained reliability figures (Alphas) range from 0.60 to 0.81 for the various job satisfaction dimensions. These findings provide support for the internal consistency of the sub-scales, so we can state that the scale of the survey questions used in the analysis was acceptable (Table 8).

**Table 8:** Reliability analysis of scales

Factor ID	Factor Name	Cronbach’s Alpha (CA)	Overall Items	Items	Average Item Score
Factor 1	Benefits and Salary	0.74	4	11, 20, 28, 33	2.27
Factor 2	Management’s attitude	0.67	4	14, 19, 24, 29	2.51
Factor 3	Supervision	0.81	4	3, 12, 21, 30	4.66
Factor 4	Communication	0.60	3	18, 26, 36	3.71
Factor 5	Nature of work	0.61	3	17, 27, 35	4.48
Factor 6	Colleagues Support	0.66	2	7, 25	4.96
**Overall Satisfaction**		**0.81**	**20**		3.76

## Confirmatory factor analysis

**Fig. 1:**
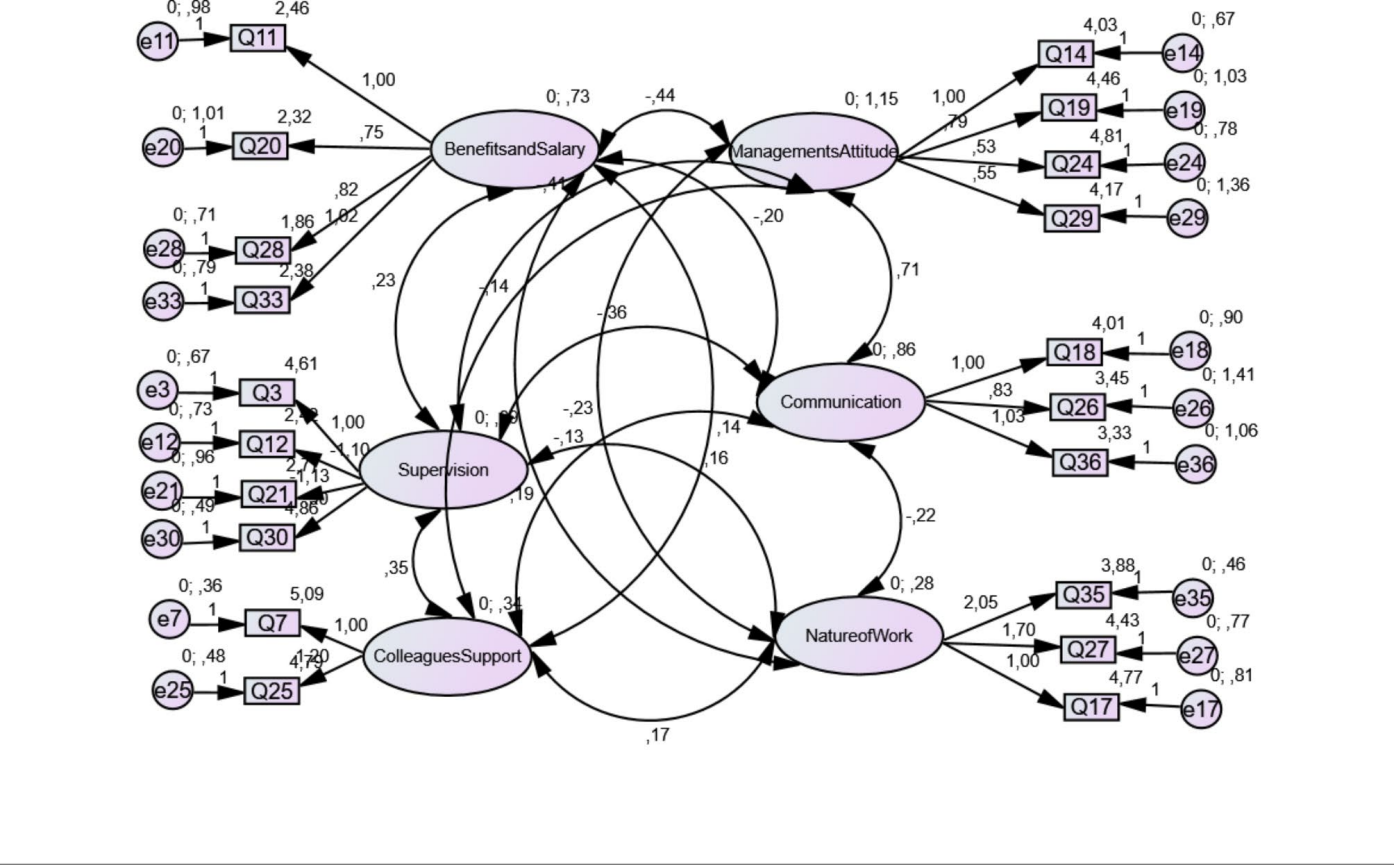
Result of confirmatory factor analysis (CFA)

Confirmatory Factor Analysis **(**CFA) is a statistical technique used to evaluate the measurement models that represent hypotheses about relations between indicators and factors. The CFA assessed the fit of the six-factor structure and the model fitted the data well as defined from the SRMR, RMSEA, CFI and IFI values that were equal to 0.050 (≤ 0.800), 0.055 (≤ 0.800), 0.906 (≥ 0.900) and 0.906 (≥ 0.900) respectively. It was suggested that the fitting optimization index was acceptable and the structure of the model was designed reasonably (Fig. 1).

## Discussion

To sum up the discussion, the basic purpose of this study was to validate Spector’s JSS instrument and develop a valid, short and reliable instrument that can measure employee job satisfaction for public hospitals in Athens, Greece. There were 3,278 responses received from the employees of thirteen different hospitals. Factor analysis was conducted due to anticipated dimensionality of factors that are involved in measuring job satisfaction. The values of Cronbach’s Alpha coefficients were computed in order to assess the internal consistency reliability.

Overall, the job satisfaction scale developed in this research illustrates valid and reliable measures for assessing hospital employees’ satisfaction levels with their work. Yet in reality, job satisfaction is a complex multidimensional concept. The study is based on the premise that an organization’s intellectual capital is its most important asset. For this purpose, our survey used a personalized “bottom-up” approach, which studied the properties of employees, their behavior at the workplace, motivators, dissatisfiers, and other properties of the job environment. Satisfied human resource is the most valuable asset for high productivity, commitment, efficiency, and quality of care in a healthcare organization [53]. Aiming we get answers to basic questions: “How do employees feel in their workplace? What makes them behave in the workplace the way they do? What would motivate them to perform well and according to the hospital’s goals?“ The employees are motivated (or not) to perform as they do because of a combination of internal and external factors, which should be investigated, measured and improved as much as possible.

The statistical analyses identified six predominant components to quantify job satisfaction, namely Benefits and Salary (F1), Management’s attitude (F2), Supervision (F3), Communication (F4), Nature of work (F5), Colleagues Support (F6). Meanwhile, among the affecting factors of job satisfaction, monetary benefits have the most influence, relationships with superiors and colleagues, training and enhancement of employee skills, the perceived fairness of the promotion system, the quality of the working conditions, and a sense of belonging are vital to the development of job satisfaction.

An important strength of this study is that a short JSS questionnaire was developed for healthcare organizations that can be used much more widely in a rapidly changing environment. This newly developed questionnaire will prove very useful in providing continuous feedback to top management as well as health policy makers regarding the level of job satisfaction. Such feedback provided by the existing health workforce will immediately alert them to any adverse working conditions that present themselves as factors leading to job dissatisfaction.

In Greece, the results of this study are important in terms of determining factors that should be considered for success within organizations. This research is valuable because it has both a practical and humanitarian application, as it gives a better understanding of employee satisfaction which in turn will lead to improved organizational behavior and employee attitudes that directly affect the improvement of health quality. Gaining employee’s commitment to their organization’s goals is believed to unlock their potential and achieve heightened levels of performance. Opposite results can lead employees to dissatisfaction or tend to lose interest in their work, higher levels of burnout and stress, absenteeism, intention to quit, and consequently suboptimal healthcare delivery and poor clinical outcomes [54]. Managers of health services organizations in cooperation with the Ministry of Health (MoH) must elicit cooperation and performance of the employees to ensure the quality of care and the morbidities and mortalities may be improved undoubtedly. Most researchers agree that employees with high job satisfaction levels have improved mental and physical health, job involvement, and improved quality of life. Eliciting such commitment from employees is not easy to obtain especially under uncertain or difficult working conditions [55–58].

More than ever, due to the globalization evolution of the Covid-19 pandemic, health systems need satisfied employees who can cope with very difficult conditions, refine health care services, and up surging the level of patient satisfaction. The study of job satisfaction is gaining more and more importance with the passage of time because of its nature and impact on society. The need to understand employee satisfaction resurfaces as everyone understands that they serve the ultimate human good, health [59].

## Conclusion

In total, this study applied quantitative methods to determine factors affecting job satisfaction. So, is important in terms of determining the specific factors that should be considered for job satisfaction, organizational engagement, managerial success, and high performance within hospitals. A short 20-item study for all healthcare staff can benefit hospitals to monitor employee satisfaction across all levels without overburdening employees and analysts with multiple or fielding several non-comparable types of research.

The findings suggest that effective communication and support from managers or supervisors to employees or among employees themselves will reduce stress and conflicts in the workplace. Additionally, it can be recommended that employee empowerment and training, collaboration in teamwork, and a systematic approach regarding innovative types of promotional opportunity, recognition, reward, and evaluation of hospital staff can lead to better results and benefits employees, quality of patient care, and healthcare organizations. Consequently, we believe that empowerment of management, achievement, promotion and evaluation should significantly improve job satisfaction respectively. This study showed that obtained factors are aligned with the findings of the prior studies in the literature [60, 61].

The results of this study should not be generalized extensively since the participants of the study come from a single geographical region of the country, only in hospitals in Athens, Greece. Nevertheless, the sample cannot be characterized as homogenous due to the fact that participants were working in different departments in the hospitals, so they deal with different tasks and procedures. Therefore, the findings and related conclusions may not be able to be generalized and compared with the rest regions of the country.

## Data Availability

The data can be accessible from the corresponding author when the Ethics Committee of the National and Kapodistrian University of Athens and the Scientific Council of Primary Health Care of the 1st Regional Health Authority of Attica provide data access permission.
