# Correction: Determining dimensions of job satisfaction in healthcare using factor analysis

**DOI:** 10.1186/s40359-022-01026-w

**Published:** 2022-12-23

**Authors:** Dimitris Karaferis, Vassilis Aletras, Dimitris Niakas

**Affiliations:** 1 Department of Health Economics, Medical School, National and Kapodistrian University of Athens, 75, M. Assias Street, 11527 Athens, Greece; 2 Department of Business Administration, University of Macedonia, Thessaloniki, Greece

**Correction to: BMC Psychology (2022) 10:240** 10.1186/s40359-022-00941-2

Following publication of the original article [1], the authors flagged that the article had published with an error in Table 3; in the 'Period of research' part of the table, the symbol ''X' (denoting 'research conduct') had been erroneously included in every cell of the table. The table has now been corrected in the published article and the corrected table may be seen in this erratum.

**Table 3:** The population of research per hospital and category. Distributed Questionnaires and Response Rate

Hospital	Period of research						Professional category										
	2019			2020			Doctors		Nurses		Other health professionals		Overall sample		Number οf beds*	Number οf patients*	Days of hospitalization*
	3Q	4Q	1Q	2Q	3Q	4Q	( n)	( %)	( n)	( %)	( n)	( %)	( n)	( %)			
H 01	X	X	X				108	13.45%	132	7.60%	43	5.82%	283	8.64%			
H 02	X	X	X	X			168	20.92%	290	16.71%	158	21.38%	616	18.80%			
H 03			X	X	X	X	29	3.61%	99	5.70%	74	10.01%	202	6.17%			
H 04		X	X	X	X		90	11.21%	210	12.10%	64	8.66%	364	11.11%			
H 05	X	X	X				59	7.35%	89	5.13%	62	8.39%	210	6.41%			
H 06			X	X	X	X	51	6.35%	122	7.03%	69	9.34%	242	7.39%			
H 07		X	X	X			61	7.60%	181	10.43%	39	5.28%	281	8.58%			
H 08		X	X	X			87	10.83%	90	5.18%	32	4.33%	209	6.38%			
H 09		X	X	X			36	4.48%	119	6.85%	57	7.71%	212	6.47%			
H 10		X	X	X			45	5.60%	111	6.39%	50	6.77%	206	6.29%			
H 11		X	X				29	3.61%	136	7.83%	36	4.87%	201	6.14%			
H 12		X	X	X	X		32	3.99%	132	7.60%	38	5.14%	202	6.17%			
H 13			X				8	1.00%	25	1.44%	17	2.30%	50	1.53%			
(1) Hospitals of research						(n = 13)	803	24.50%	1736	52.96%	739	22.54%	3278	100%	6511	438,745	1,443,660
(2) Hospitals in 1st regional health authority of attica						(n = 27)	6277**	31.71%	8174**	41.29%	5345**	27.00%	19,796**	100%	8860	567,817	1,912,488
(3) Hospitals in the national health system						(n = 127)									36,441	2,160,596	7,343,348
(4) Percentages of hospitals of research/hospitals of attica						= (1)/(2)			12.79%		21.24%		13.83%		16.56%	73.49%	77.27%
(5) Percentages of hospitals of research/NHS						= (1)/(3)									17.87%	20.31%	19.66%
(6) Distributed questionnaires of research							1,000	25.00%	2000	50.00%	1000	25.00%	4,000	100%			
(7) Response rate						= (1)/(6)		80.30%		86.80%		73.90%		81.95%			

*Data of year 2020, **Data of year 2016.

X = research conduct.

The publisher thanks you for reading this erratum and apologizes for any inconvenience caused.

